# Prevalence of Diabetes and the Relationship Between Wealth and Social Demographic Characteristics Across 6 Low-and-Middle Income Countries

**DOI:** 10.5539/gjhs.v16n4p22

**Published:** 2024-03-25

**Authors:** Gifty Marfowaa, Jennifer A. Campbell, Sneha Nagavally, Aprill Z. Dawson, Rebekah J. Walker, Leonard E. Egede

**Affiliations:** 1 Center for Advancing Population Science, Medical College of Wisconsin, Milwaukee, WI, USA; 2 Division of General Internal Medicine, Department of Medicine, Medical College of Wisconsin, Milwaukee, WI, USA

**Keywords:** wealth, income, diabetes, global

## Abstract

**Background:**

As the global burden of diabetes persists, research is needed to understand the role of wealth and correlates of diabetes across regions of the world. The purpose of this study is to examine the prevalence and role of wealth and diabetes across 6 low- and middle- income countries while also accounting for independent correlates of diabetes by country.

**Methods:**

Data from the Study on Global Ageing and Adult Health (SAGE), SAGE Wave 1 was used. Self-reported diabetes status was the primary dependent variable and wealth quintile, number of dwelling characteristics and possession of a set of assets, was the independent variable. Logistic regression models examined the relationship between wealth and presence of diabetes across 6 countries with the highest wealth quintile, quintile 1, serving as the reference group.

**Results:**

Sample size by country included Ghana N = 5573, South Africa N = 4227, Russia N = 4947, Mexico N = 5448, India N = 12198, and China N = 15050. Average age across country ranged from 49 to 63 years of age. Prevalence of diabetes across country included 3.4% and 9.2% for Ghana and South Africa, respectively. In Russia, 8.3%; Mexico, 18.1%; India, 4.9%; and China, 5.9% of the sample reported having diabetes. In the adjusted logistic model, wealth was associated with higher odds of diabetes in Ghana (OR 2.26; CI 1.28; 4.13), South Africa (OR 4.57; CI 2.25; 10.32), Mexico (OR 2.00; CI 1.14; 3.60), India (OR 2.45; CI 1.60; 3.86), and China (OR 2.16; CI 1.62, 2.93).

**Conclusions:**

These findings add to the growing body of evidence in our understanding between wealth and diabetes. As diabetes persists as a leading cause of death globally, future work should focus on mechanisms underlying the relationship between wealth and diabetes while also developing interventions to mitigate his burgeoning disease affecting communities across low- and middle-income countries.

## 1. Introduction

Diabetes is the 7th leading cause of death worldwide, affecting 537 million people who are 20-79 years of age. (International Diabetes Federation [IDF], 2021; World Health Organization [WHO], 2022; Lin et al., 2020; van Dieren et al., 2010). The incidence and prevalence of diabetes continues to increase on a global level, exerting significant economic cost at the individual and societal level (IDF, 2021; WHO, 2022; Lin et al., 2020; van Dieren et al., 2010; Cho et al., 2018; King, Aubert, & Herman, 1998). According to the International Diabetes Federation (IDF) 10th edition of the diabetes atlas released in 2021, the geographic profile of age adjusted diabetes prevalence shows that the highest prevalence for adults 20-79 years of age is experienced by the Middle East and North Africa (MENA) region at 18%, followed by the North American and Caribbean region at 11.9%, South-East Asia at 10%, Western Pacific at 9.9%, South and Central America at 8.2%, Europe at 7%, and Africa at 5.3% (IDF, 2021). While family history and genetics may increase risk for developing diabetes, lifestyle factors such as poor diet, limited physical activity, and being overweight are key risk factors (WHO, 2022). Recent evidence suggests an epidemiologic transition, shifts in the leading causes of death from communicable to other causes of death, may be a key factor underlying the rise in diabetes, particularly for low- and middle-income countries. Epidemiological transitions represent the complex shifts in health and disease and how these patterns in turn impact the demographic and economic profile of populations (Omran, 2005; Mattei et al., 2015). While there are many determinants of epidemiological transitions, wealth is an important factor as it increases access to resources to support infrastructure for improving population health (Omran, 2005; Mattei et al., 2015).

Wealth as a factor associated with increasing diabetes prevalence has gained attention more recently as epidemiological transitions have occurred across regions of the world, shifting the leading causes of death to non-communicable diseases such as diabetes (Alcorn & Ouyang, 2012; Williams et al., 2018; Klautzer, Becker, & Mattke 2014; Tanaka, Gjonça, & Gulliford, 2011). For example, a recent study examining the relationship between wealth and diabetes prevalence across 29 low- and middle-income countries found that higher household wealth was associated with increased diabetes risk across lower income countries but not in middle income countries (Seiglie et al., 2020). Other studies have examined the relationship with wealth and diabetes at country specific levels across low-and middle-income countries as well as high income countries (Tanaka, Gjonça, & Gulliford, 2011; Mutyambizi et al., 2019). In the United Kingdom for example, lower wealth was associated with higher risk of diabetes among the older population (Tanaka, Gjonça, & Gulliford, 201). Whereas in South Africa, diabetes prevalence is found concentrated within groups with higher wealth (Mutyambizi et al., 2019).

As diabetes prevalence continues to rise across regions of the world, understanding the relationship between wealth and diabetes is a critical next step for the development of global efforts to mitigate the impact of diabetes (WHO, 2016; WHO, 2010). Specifically, the World Health Organization (WHO) and the IDF support the development of multisectoral responses across regions of the world to combat diabetes with specific focus on care and control, national plans and strategies to reduce impact, and high-quality research (WHO, 2018; IDF, 2023). In support of these multisectoral responses, understanding the relationship between wealth and diabetes across geographic regions may better inform multisectoral approaches. This paper therefore aims to extend the evidence base in three keyways. First, this paper will examine prevalence of diabetes across six countries that represent five IDF regions including 1) Africa; 2) Europe; 3) North America and Caribbean; 4) South-East Asia; and 5) Western Pacific. Second, this paper will examine the relationship between wealth and diabetes and if this relationship varies across the 6 countries. Finally, this paper will examine correlates of diabetes across social demographic factors by countries.

## 2. Method

### 2.1 Data

This study used data from the WHO Study on Global Ageing and Adult Health (SAGE) (WHO, 2024; Kowal et al., 2012). SAGE is an international longitudinal dataset that collects health surveillance data from national representative samples across six countries: Ghana, South Africa, Russian Federation, Mexico, India, and China, each country included in the WHO SAGE study represents the partnered countries with the WHO for SAGE (Kowal et al., 2012). According to WHO for SAGE, these six countries are core SAGE countries selected as such for their broad representation across geographic regions capturing economic diversity and health transitions (Kowal et al., 2012). SAGE collects data from adults aged 18 years and older, placing emphasis on adults who are age 50 and older across participating countries (Kowal et al., 2012). Participant responses from SAGE Wave 1 individual data files were used for each participating country (Kowal et al., 2012). SAGE Wave 1 was conducted during the time period of 2007 to 2010 and captured both individual and household level information. Wave 1 represents the only wave with current data available for all 6 countries (WHO, 2024). Ghana, South Africa, Russian Federation, India, and China utilized a multistage cluster sampling strategy. In Mexico, all 32 federal states were included in the household section (Kowal et al., 2012). Detailed sample size by country, response rates, representativeness level, and country validity have been published and described in detail by the WHO for SAGE in Kowal et al. (2012). Wave 1 measures include socio-demographics, work history and benefits, health status, performance tests and biomarkers, risk factors and preventive health behaviors, chronic conditions, social cohesion, and health care utilization. Individual response rate in China was 93%, 80% in Ghana, 68% in India, 51% in Mexico, 83% in Russia and 77% in South Africa. The total number of individual respondents from China was 15,050; 5,573 in Ghana, 12,198 from India, 5,448 from Mexico, 4,947 in Russia and 4,227 in South Africa.

### 2.2 Measures

#### 2.2.1 Dependent Variable

Diabetes status served as the dependent variable. Diabetes status was self-reported based on responses to any of the following questions: 1) Have you ever been diagnosed with diabetes (high blood sugar) (not including diabetes associated with a pregnancy); 2) Have you been taking insulin or other blood sugar lowering medications; 3) Have you been following a special diet, exercise regimine or weight control program for diabetes during the last 2 weeks? (As recommended by health professional). Diabetes type cannot be determined in this dataset, however given that 90-95% of all diabetes cases worldwide represent type 2, it can be assumed that the majority of participants in this study have type 2 diabetes (WHO, 2022).

#### 2.2.2 Independent Variables

Wealth served as the independent variable derived from household assets, characteristics of household dwelling, and self-reported consumption and income, available in the dataset as ‘income quintile’ for each country (Kowal et al., 2012; Fernández-Niño et al., 2019). Quintiles range from 1-5 with 1 representing the lowest quintile and 5 representing the highest (Kowal et al., 2012). The income quintiles are created based a number of dwelling characteristics and possession of a set of assets (Ferguson, Tandon, & Gakidou, 2003).

#### 2.2.3 Social Demographics

Social demographics included age (continuous), gender (male, female), marital status (categorized as ‘Single’, ‘with Partner’), education (‘No formal education’, ‘Less than High School’, ‘High School or beyond’), area of residence (‘Urban’, ‘Rural’), work status (‘Working’ vs. ‘Not-Working’), and social cohesion (‘Yes’, ‘No’) to capture neighborhood characteristics of the sample. Social cohesion was measured using the following three components: 1) Trust, 2) Safety Perception, and 3) Social Network. Trust was captured by asking participants about trust in neighbors with response types as ‘To a very great extent’, ‘To a great extent’, ‘Neither great nor small extent’, ‘To a small extent’, ‘To a very small extent’. The variable was dichotomized by grouping ‘To a very great extent’,’ To a great extent’ responses to ‘Yes’ and all other response types as ‘No’. Safety perception was determined using the following items 1) Feel safe at home? 2) Feel safe on street? With responses as ‘completely safe’, ‘very safe’, ‘moderately safe’, ‘slightly safe’ and ‘not safe at all’. The above two variables were dichotomized by grouping responses ‘completely safe’, ‘very safe’ as to being ‘safe’ and other response types grouped into ‘not safe’ category. The third survey item for safety included 3) Victim of violent crime last 12 months? Response type as ‘Yes’ or ‘No’. We consolidated items 1, 2, 3 to a single continuous variable ranging from 0 (being low) to 3 (being high). Further simplified by generating a categorical variable with low (0) vs. medium/high (1-3) categories. Social Network was measured using marital status, attending religious services, participating in clubs, and trusting other people. Each of the items were categorized and further generated a continuous variable ranging from 0 to 4 and simplified it to a categorical variable as ‘No (0)’ vs ‘Yes (1-4)’.

### 2.3 Statistical Analysis

Descriptive statistics to determine frequencies were used to understand social demographic differences between the regions/countries. The relationship between wealth and presence of diabetes in each region/country was assessed using logistic regression models. Specifically, generalized linear regression models with family defined as binomial and link type as logit to account for dichotomized outcomes and to implement a distribution on the error terms for the populations from China, Ghana, India, Mexico, Russia, and South Africa. Models were stratified by country. First, unadjusted analyses were run with wealth as the primary independent variable and diabetes as the outcome. Then models were adjusted for social demographic characteristics (age, gender, marital status, education, area of residence, work status, and social cohesion). We used R version-4.0.0 to perform our statistical analysis and statistical significance was set at p<0.05.

## 3. Results

Table 1 summarizes the sample characteristics. Sample size by country included Ghana N = 5573, South Africa N = 4227, Russia N = 4947, Mexico N = 5448, India N = 12198, and China N = 15050. Mean age across each country was 60 years for Ghana, 60 years for South Africa, 62 years for Russia, 63 years for Mexico, 49 years for India, and 60 years for China. Looking at prevalence of diabetes by country, Ghana & South Africa, prevalence was 3.4% and 9.2%, respectively. In Russia, 8.3% of the sample reported having diabetes. In Mexico, 18.1% of the sample population reported having diabetes. In India, 4.9% of the sample reported having diabetes. In China, 5.9% of the sample reported having diabetes.

Table 2 shows the unadjusted logistic models for the relationship between wealth and odds of diabetes by country. Ghana had statistically significantly higher odds of diabetes for quintile 4 (OR 2.35; CI 1.39, 4.14) and quintile 5 (OR 3.70; CI 2.26, 6.35) compared to those in quintile 1. South Africa had statistically significantly higher odds of diabetes for quintile 3 (OR 3.19; CI 2.07, 5.07), quintile 4 (OR 3.95; CI 2.60, 6.22), and quintile 5 (OR 4.04; CI 2.66, 6.35) compared to quintile 1. In Russia, there was no statistically significant relationship between wealth and diabetes. Mexico had statistically significantly higher odds of diabetes for quintile 3 (OR 1.55; CI 1.13, 2.13) only, compared to quintile 1. India had statistically significantly higher odds of diabetes for quintile 3 (OR 2.26; CI 1.55, 3.36), quintile 4 (OR 3.57; CI 2.51, 5.18), and quintile 5 (OR 4.81; CI 3.43, 6.92) compared to quintile 1. China had statistically significantly higher odds of diabetes for quintile 2 (OR 1.49; CI 1.13; 1.97), quintile 3 (OR 2.18; CI 1.68; CI 2.83) quintile 4 (OR 2.47; CI 1.92; 3.19) and quintile 5 (OR 2.64; CI 2.06; 3.41) compared to those in the lowest quintile.

Table 3 shows the logistic model for the relationship between wealth and diabetes adjusted for social demographic characteristics (age, gender, marital status, education, area of residence, work status, and social cohesion) by country. Individuals in Ghana had a statistically significantly higher odds of diabetes for quintile 4 (1.88; 1.07, 3.42) and quintile 5 (OR 2.26; CI 1.28, 4.13). Individuals in South Africa had a statistically significantly higher odds of diabetes for quintile 3 (OR 4.07; CI 2.03, 9.09), quintile 4 (OR 4.89; CI 2.47, 10.84), and quintile 5 (OR 4.57; CI 2.25, 10.32). In Russia, there was no statistically significant higher odds of diabetes in any of the quintiles. Individuals in Mexico had a statistically significantly higher odds of diabetes for quintile 2 (OR 2.06; CI 1.19, 3.64), quintile 3 (OR 2.04; CI 1.17, 3.66), and quintile 5 (OR 2.00; CI 1.14, 3.60). Individuals in India had a statistically significantly higher odds of diabetes for quintile 3 (OR 1.78; CI 1.14, 2.84), quintile 4 (OR 2.34; CI 1.54, 3.67), and quintile 5 (OR 2.45; CI 1.60, 3.86). Individuals in China had a statistically significantly higher odds of diabetes for quintile 2 (OR 1.57; CI 1.15, 2.17), quintile 3 (OR 1.83; CI 1.36, 2.48), quintile 4 (OR 2.23; CI 1.67, 3.01), and quintile 5 (OR 2.16; CI 1.62, 2.93).

Table 3 also shows the correlates of diabetes across social demographic characteristics by region. In Ghana, rural area of residence was statistically significant with lower odds of diabetes (OR 0.56; CI 0.39, 0.81) compared to urban. Unemployment was significantly associated with higher odds of diabetes (OR 2.14; CI 1.52, 3.03) compared to being employed. Similarly, in South Africa, rural area of residence was significantly associated with lower odds of diabetes (OR 0.44; CI 0.29, 0.66) and unemployment was significantly associated with higher odds of diabetes (OR 2.58; CI 1.72, 3.95). In Russia, social demographic characteristics associated with higher odds of diabetes (OR 1.02; CI 1.00, 1.03), women (OR 2.00; CI 1.49, 2.73), and unemployment (OR 2.00, CI 1.43, 2.83). In Mexico, older age was the only social demographic characteristic associated with higher odds of diabetes (OR 1.02; CI 1.01, 1.03) and rural area of residence was associated with lower odds of diabetes (OR 0.50; CI 0.32, 0.76). For India, social demographic characteristics associated with increased odds of diabetes included older age (OR 1.03; CI 1.02, 1.04), greater than a high school education (OR 1.74; CI 1.36, 2.21) less than a high school education (OR 1.58; CI 1.01, 2.43) compared to no education, respectively, and unemployment (OR 1.29; CI 1.00, 1.65). Women in India had significantly lower odds of diabetes compared to men (OR 0.63; CI 0.47, 0.82), and rural area of residence (OR 0.49; CI 0.39, 0.62) compared to urban area of residence. For China, older age (OR 1.04; CI 1.03, 1.06), having a partner compared to being single (OR 1.31; CI 1.05, 1.64), and unemployment compared to being employed (OR 1.65; CI 1.32, 2.07) were all statistically significantly related to higher odds of diabetes. Living in a rural area of residence compared to an urban area was statistically significantly related to lower odds of diabetes in China (OR 0.45; CI 0.36, 0.55).

## 4. Discussion

Overall, this study examining the relationship between wealth and odds of diabetes across 6 countries found prevalence of diabetes varied across countries with the highest prevalence seen in Mexico with 18.1% prevalence. Prevalence varied between the two African countries with a 9% prevalence in South Africa and a 3.4% prevalence in Ghana. In Russia, diabetes prevalence was 8.3%, and was 5.9% for China, and 4.9% in India. After adjusting for social demographic factors, findings showed a significant gradient between wealth and diabetes for Ghana and India. However, this gradient was not consistent for all countries. For example, in China, there was a slight gradient for wealth across quintiles 2, 3, and 4, however this relationship decreased for quintile 5. For Mexico, wealth for quintiles 2 and 3 were significantly associated with higher odds of diabetes but was not significant for quintile 4, and significance decreased for quintile 5. For South Africa, wealth was significantly associated with 4-fold increased odds of diabetes for quintiles 3, 4, and 5 but there was no gradient. In Russia, wealth was not statistically significantly related to diabetes across any quintile.

Correlates of diabetes also varied across regions. Living in a rural residence was significantly associated with lower odds of diabetes in Ghana and South Africa, Mexico, India, and China. Unemployment was consistently associated with higher odds of diabetes across all countries except for Mexico. Older age was significantly associated with higher odds of diabetes for Russia, Mexico, India, and China. Women had a significantly higher odds of diabetes in Russia, however had significantly lower odds of diabetes in India. Individuals who reported having a partner, compared to being single, was only significantly related to higher odds of diabetes in China; and having any education was significantly associated with higher odds of diabetes in India only.

Overall, existing evidence shows that there is a clear gradient between country level wealth and diabetes prevalence, with a recent study of 29 low and middle income, upper middle income, and high-income countries showing that across world bank income groups, diabetes prevalence increased with increasing country wealth (Seiglie et al., 2020). The current findings add to the body of literature by providing country level data for the relationship between wealth and diabetes across 6 countries. Specifically, in China, evidence for the relationship between wealth and diabetes has been mixed (Wu et al., 2017). A systematic review evaluating the relationship between socioeconomic status and diabetes in China, Hong Kong, and Taiwan found that the relationship between income and diabetes was inconsistent in the literature (Wu et al., 2017). However, more recently, a study examining the relationship between socioeconomic status and diabetes in China, found that after adjusting for demographic factors and family history of diabetes, household income was significantly associated with prevalent diabetes in a prospective cohort of Chinese adults (Wu et al., 2019). In India, recent evidence from a population-based study shows diabetes prevalence as being 2.9%, with household wealth being significantly associated with diabetes in the study population (Corsi and Subramanian, 2019). Results from the NATION study conducted in the Russian Federation found in 2016 the diabetes prevalence to be 5.4%, with significantly higher prevalence found among rural populations compared to urban populations, however this study did not specify wealth or income as factor controlled or associated with diabetes in the population (Dedov et al., 2016). Evidence for South Africa shows that diabetes prevalence varies by wealth among its population, however individuals with diagnosed diabetes are represented amongst the highest wealth quintiles in the country (Mutyambizi et al., 2019). Similarly in Ghana, at a population level, households with greater wealth are shown to have higher diabetes prevalence compared to those with lower wealth (Gatimu, Milimo, & Sebastian, 2016). For Mexico, Gross Domestic Product (GDP) has not only been shown to be associated with diabetes but is also associated with diabetes mortality such that the higher the GDP over time in Mexico, the higher the diabetes mortality rate (Soto-Estrada et al., 2018).

The findings of this study show that the relationship between wealth and diabetes is not consistent across countries, and correlates of diabetes also vary across country. This differential relationship between wealth, social demographic correlates, and diabetes across these 6 countries suggests the need for tailoring of global efforts to account for the unique drivers of diabetes across the world. Specifically, focusing on prevalence by geographic profile may not be adequate to address diabetes at a global level. For example, as the IDF provides prevalence estimates for each region, demonstrating the lowest prevalence of diabetes in the Africa region with 5.3% 1, the findings presented here show that while Africa may have the lowest diabetes prevalence of the IDF regions according to the IDF Diabetes Atlas, South Africa within this region has a 4- fold increased odds of diabetes for individuals in wealth quintiles 3, 4, and 5. Additionally, as reported by the IDF Diabetes Atlas, the North American and Caribbean region has a diabetes prevalence of 11.9% (IDF, 2021). The findings of this study show that Mexico within this region has a diabetes prevalence of 18% and odds of diabetes decreases as wealth increases. Taken together, the data presented here extend the data of the IDF by highlighting diabetes prevalence and the association between wealth and diabetes across countries within represented IDF regions with variation in correlates of diabetes across countries.

## 5. Limitations

While this study is strengthened by examining the relationship between wealth and diabetes across 6 countries there are several limitations that need to be taken into account. First, this data represents a cross-sectional analysis and so these results cannot speak to causality. Future work should consider the longitudinal examination between wealth and odds of diabetes as more years of data for all 6 countries become available. Secondly, the data presented here is self-report and may be subject to some recall bias and or misclassification, however evidence suggests that self-report of chronic disease conditions, such as diabetes, has low recall bias. Additionally, reverse causality may explain the association between diabetes and unemployment in some countries, however this dataset did not specify unemployment due to retirement versus not being able to find work as a working age adult seeking employment. Finally, measuring wealth across countries may present some level of variability, future work should also consider using a wealth index that can be harmonized across countries to minimize bias.

## 6. Conclusions

This study examined the relationship between wealth and diabetes across 6 countries. Findings show that prevalence of diabetes varies across countries and wealth is associated with diabetes across countries, with an inconsistent gradient between wealth and diabetes existing across countries. Additionally, this study shows that correlates of diabetes vary by country suggesting the need for a unique and tailored approach to address diabetes across world regions. As multisectoral approaches are developed to combat the global burden of disease, these findings add to the growing body of evidence in our understanding between wealth and odds of diabetes. Future work should focus on mechanisms underlying the relationship between wealth and diabetes while also developing tailored interventions that account for the unique correlates of diabetes to mitigate this burgeoning disease affecting communities across low- and middle-income countries.

## Figures and Tables

**Table 1.** Sample Characteristics by Country

	Africa		Europe	North America and Caribbean	South-East Asia	Western Pacific
	Ghana (N=5573)	South Africa (N=4227)	Russia (N=4947)	Mexico (N=5448)	India (N=12198)	China (N=15050)
Self-reported Diabetes	3.4%	9.2%	8.3%	18.1%	4.9%	5.9%
**Independent Variable Wealth**						
Quintile 1 (lowest income quintile)	19.4%	19.3%	17.3%	20.3%	17.4%	19.0%
Quintile 2	19.7%	19.6%	19.1%	20.1%	19.1%	19.8%
Quintile 3	19.8%	18.9%	20.1%	19.6%	19.1%	20.0%
Quintile 4	20.2%	20.7%	20.7%	20.0%	21.0%	20.6%
Quintile 5 (highest income quintile)	20.6%	21.3%	22.7%	19.8%	23.2%	20.4%
**Social Demographic Characteristics**						
Mean Age (sd)	60.2(14.1)	60.3(12.4)	62.4(13.0)	63.7(14.3)	49.9(16.8)	60.5(11.9)
**Gender**						
Female	49.4%	57.4%	64.4%	61.7%	61.4%	53.4%
**Marital Status**						
w/Partner	60.9%	53.0%	57.1%	63.4%	77.5%	83.4%
**Level of Education**						
No formal education	82.2%	54.4%	10.3%	53.7%	59.3%	64.1%
<High School	9.2%	38.7%	52.9%	42.6%	32.1%	30.2%
> = High School	8.5%	6.8%	36.7%	3.6%	8.5%	5.5%
**Area of Residence**						
Rural	59.0%	33.4%	25.0%	23.5%	74.3%	50.9%
**Work Status**						
Not Working	26.7%	67.7%	62.5%	46.5%	35.8%	51.1%
**Social Cohesion**						
Yes	55.2%	24.7%	24.1%	36.4%	45.3%	75.5%

**Table 2.** Unadjusted Logistic Model of Wealth and Odds of Diabetes by Country

	Africa		Europe	North America and Caribbean	South-East Asia	Western Pacific
	Ghana N = 5,083	South Africa N = 4,005	Russia N = 4,289	Mexico N = 2,630	India N = 11,152	China N = 14,480
	OR (CI)	OR (CI)	OR (CI)	OR (CI)	OR (CI)	OR (CI)
**Wealth**						
Quintile 2	0.83 (0.42, 1.63)	1.61 (0.99, 2.65)	0.96 (0.67, 1.36)	1.35 (0.99, 1.86)	1.28 (0.84, 1.96)	**1.49** ** **(1.13, 1.97)**
Quintile 3	1.36 (0.75, 2.51)	**3.19** *** **(2.07, 5.07)**	1.28 (0.92, 1.79)	**1.55** ** **(1.13, 2.13)**	**2.26** *** **(1.55, 3.36)**	**2.18** *** **(1.68, 2.83)**
Quintile 4	**2.35** ** **(1.39, 4.14)**	**3.95** *** **(2.60, 6.22)**	0.81 (0.57, 1.17)	1.16 (0.84, 1.61)	**3.57** *** **(2.51, 5.18)**	**2.47** *** **(1.92, 3.19)**
Quintile 5	**3.70** *** **(2.26, 6.35)**	**4.04** *** **(2.66, 6.35)**	0.78 (0.55, 1.11)	1.19 (0.85, 1.65)	**4.81** *** **(3.43, 6.92)**	**2.64** *** **(2.06, 3.41)**

*Note.* ***p < .001; **p < .01; *p < .05. OR = Odds Ratio. CI = Confidence Interval.

**Table 3.** Adjusted Logistic Multivariable Model of Wealth and Odds of Diabetes by Country

	Africa		Europe	North America and Caribbean	South-East Asia	Western Pacific
	Ghana N = 4,816	South Africa N = 2,301	Russia N = 3,776	Mexico N = 1,240	India N = 7,109	China N = 11,977
	OR (CI)	OR (CI)	OR (CI)	OR (CI)	OR (CI)	OR (CI)
**Wealth**						
Quintile 2	0.81 (0.41, 1.61)	2.15 (1.01, 4.97)	0.94 (0.64, 1.38)	**2.06** * **(1.19, 3.64)**	1.14 (0.70, 1.88)	**1.57** ** **(1.15, 2.17)**
Quintile 3	1.24 (0.67, 2.33)	**4.07** *** **(2.03, 9.09)**	1.35 (0.94, 1.94)	**2.04** * **(1.17, 3.66)**	**1.78** * **(1.14, 2.84)**	**1.83** *** **(1.36, 2.48)**
Quintile 4	**1.88** * **(1.07, 3.42)**	**4.89** *** **(2.47, 10.84)**	0.99 (0.66, 1.49)	1.71 (0.97, 3.10)	**2.34** *** **(1.54, 3.67)**	**2.23** *** **(1.67, 3.01)**
Quintile 5	**2.26** ** **(1.28, 4.13)**	**4.57** *** **(2.25, 10.32)**	1.07 (0.70, 1.61)	**2.00** * **(1.14, 3.60)**	**2.45** *** **(1.60, 3.86)**	**2.16** *** **(1.62, 2.93)**
**Social Demographic Characteristics**						
**Age**	1.01 (1.00, 1.02)	1.01 (1.00, 1.03)	**1.02** * **(1.00, 1.03)**	**1.02** ** **(1.1, 1.03)**	**1.03** *** **(1.02, 1.04)**	**1.04** *** **(1.03, 1.05)**
**Gender**						
Female	1.37 (0.95, 1.99)	1.37 (0.97, 1.94)	**2.00** *** **(1.49, 2.73)**	1.04 (0.74, 1.47)	**0.63** ** **(0.47, 0.82)**	1.09 (0.93, 1.27)
**Marital Status**						
w/Partner	1.19 (0.80, 1.77)	1.07 (0.76, 1.50)	0.98 (0.75, 1.29)	1.03 (0.72, 1.48)	1.09 (0.81, 1.49)	**1.31** * **(1.05, 1.64)**
**Education**						
Educ< High School	1.54 (0.97, 2.37)	1.23 (0.87, 1.75)	1.26 (0.84, 1.92)	0.77 (0.55, 1.07)	**1.74** *** **(1.36, 2.21)**	0.98 (0.82, 1.17)
Educ> = High School	1.08 (0.63, 1.78)	1.32 (0.70, 2.37)	1.44 (0.93, 2.28)	0.44 (0.16, 0.99)	**1.58** * **(1.01, 2.43)**	1.01 (0.74, 1.36)
**Area of Residence**						
Rural	**0.56** ** **(0.39, 0.81)**	**0.44** *** **(0.29, 0.66)**	0.82 (0.60, 1.11)	**0.50** ** **(0.32, 0.76)**	**0.49** *** **(0.39, 0.62)**	**0.45** *** **(0.36, 0.55)**
**Work Status**						
Not-Working	**2.14** *** **(1.52, 3.03)**	**2.58** *** **(1.72, 3.95)**	**2.00** *** **(1.43, 2.83)**	1.35 (0.96, 1.90)	**1.29** * **(1.00, 1.65)**	**1.65** *** **(1.32, 2.07)**
**Social Cohesion**						
Yes	0.73 (0.52, 1.00)	1.05 (0.73, 1.49)	1.01 (0.75, 1.33)	0.96 (0.69, 1.33)	1.04 (0.83, 1.30)	0.92 (0.77, 1.09)

*Note.* Adjusted model covariates: Social Cohesion, Age, Gender, Marital Status, Education, Area of Residence, Work Status.

*** p < .001

** p < .01

* p < .05. OR = Odds Ratio, CI = Confidence Interval.

## Data Availability

The data that support the findings of this study are available on request.
